# Transepithelial photorefractive keratectomy: a prospective randomized comparative study between the two-step and the single-step techniques

**DOI:** 10.1038/s41433-022-02174-4

**Published:** 2022-07-21

**Authors:** Mahmoud Abdel-Radi, Mohamed Shehata, Magdi Mohammad Mostafa, Mohamed Omar M. Aly

**Affiliations:** Department of Ophthalmology, Assiut University, Assiut, Egypt

**Keywords:** Refractive errors, Education

## Abstract

**Objectives:**

To assess and compare the six-month outcome of the two-step transepithelial phototherapeutic keratectomy- photorefractive keratectomy (PTK-PRK) and the single-step transepithelial PRK for myopia and myopic astigmatism.

**Methods:**

A prospective randomized study. The study enrolled 100 eyes of 50 patients with mild to moderate myopia or myopic astigmatism stratified into two groups, PTK-PRK (*n* = 50 eyes) and single step PRK (*n* = 50 eyes). Primary outcome measures were visual acuity and manifest refraction. Secondary outcome measures were epithelial healing duration, post-PRK pain scores and 3-month postoperative haze grading.

**Results:**

Preoperative characteristics were similar in both groups (*p* value > 0.05). The mean uncorrected distance visual acuity (UDVA) at 1 week, 1 month, 3 and 6 months was significantly better in the single-step PRK group than in the two-step PTK-PRK group (*p* < 0.001). The mean manifest sphere, cylinder and spherical equivalent showed a significant difference at all follow up visits in favour of the single-step PRK (*p* value < 0.001). Epithelial healing duration was faster in single-step PRK (*p* value < 0.001). Pain scores were significantly lower following single-step PRK at 8 h, 1 day, 3 days (*p* value < 0.001) but were similar at the 7th day. Haze scores showed no statistical difference between the two groups at 3-month follow-up.

**Conclusion:**

The two transepithelial PRK techniques were effective in correcting mild to moderate myopia and myopic astigmatism. However, Single-step transepithelial PRK achieved faster visual recovery, better refractive outcome and shorter epithelial healing time with less post-PRK pain.

**Clinical trials registry:**

(Clinical Trials.gov Identifier): NCT04710082.

## Introduction

Corneal refractive surgery accounts for the majority of refractive surgeries performed to correct myopia, hyperopia and astigmatism [1]. Although LASIK is one of the commonest refractive procedures performed, the occurrence of flap related complications, corneal ectasia and dry eye have increased the popularity of flapless laser vision correction and surface ablation techniques such as small incision lenticule extraction (SMILE) and PRK [2–4].

The conventional PRK procedure involves the removal of corneal epithelium whether manually or with alcohol followed by excimer laser ablation to correct the refractive error. Manual or alcohol assisted epithelial removal was associated with some drawbacks including prolonged epithelial healing secondary to basement membrane injury or potential toxicity of alcohol, significant pain and variable degrees of stromal haze even with the use Mitomycin C [5].

Transepithelial PRK (TE-PRK, first described by Dr. Donald Johnson of Canada) was initially introduced as a two-step procedure to overcome the drawbacks of conventional PRK through the use of excimer laser phototherapeutic keratectomy PTK as a first step to remove the epithelium followed by stromal laser ablation as a second step [6, 7].

Single-step trans-epithelial PRK is relatively a new technology that involves the incorporation of both epithelial removal and stromal laser ablation in a single-step profile overcoming the less uniform ablation with limited diameter and depth in the classic PTK-PRK procedure [8].

Most of the previous studies paid particular attention to compare the transepithelial PRK procedures with the conventional manual or alcohol assisted PRK [9–13]. The aim of the current study is to focus on comparing the outcomes of the two trans-epithelial PRK techniques, the classic two-step PTK-PRK and the new single-step TE-PRK.

## Patients and methods

### Study design

A prospective comparative randomized interventional study conducted at TIBA eye center (Private practice), Assiut/Egypt.

Fifty patients were randomly classified into two groups according to the planned surgical trans-epithelial PRK technique, 25 patients planned to undergo bilateral two-step trans-epithelial PTK-PRK procedure (50 eyes) and 25 patients planned to undergo bilateral single-step trans-epithelial PRK procedure (50 eyes). Randomization was computer generated using SPSS version 26.0. Masking of the outcomes assessor was applied. (One of the authors wasn’t involved in the preoperative assessment or surgical procedures and was only responsible for postoperative assessment of all the studied outcome measures being masked about the performed TE-PRK technique).

### Patient selection

PRK candidates with myopia up to −6 dioptres and myopic astigmatism up to −4 dioptres were included with corneal thickness at the thinnest location ≥480 μm and a residual stromal bed ≥350 μm after epithelial and stromal ablation. Exclusion criteria were patients not candidate for PRK, previous corneal surgery, dry eye disease and systemic diseases such as autoimmune connective tissue disorders.

### Preoperative assessment

Ophthalmic examination was performed including uncorrected and corrected distance visual acuity (UDVA & CDVA) measurement using Snellen’s acuity chart converted to a logarithm of the minimum angle resolution (logMAR) after cessation of contact lens wear for at least two weeks before examination. Manifest and cycloplegic refractions were recorded (Auto-keratorefractometer KR-8900, Topcon). Assessment also includes slit-lamp biomicroscopy of anterior and posterior segments, intraocular pressure measurement with a calibrated Tono-pen AVIA (TPA, Reichert Inc.), Schirmer I test and Tear film break-up time (TBUT). Preoperative clinical assessment was done by the same ophthalmologist (M.S)

All patients underwent Spectral Domain Anterior segment OCT assessment (Heidelberg, GmbH, Germany) with an axial resolution of 4–7 μm and a transverse resolution of 14 μm for corneal epithelial mapping. Pentacam (Oculus GmbH, Germany) was the standard tool for Keratorefractive evaluation. Investigative assessment was done by the same ophthalmologist (M.M)

### Surgical technique

WaveLight EX-500 (WaveLight®; Alcon Laboratories, Fort Worth, TX, USA) was the device utilized for accomplishing the PRK procedure. Refractive correction in both groups was based on Wellington nomogram to achieve postoperative emmetropia in all included eyes. After sterilizing the periocular skin and eyelashes with povidone-iodine solution 10%, a drop of a preservative free local anaesthetic was instilled, and a lid speculum was inserted. A wet sponge (Merocel sponge, Medtronic Inc., Minneapolis, MN, USA) was applied to smoothly wet and cool the cornea followed by uniform drying with a dry sponge and the patient was asked to look straight-ahead at a green fixation intermittent light throughout the whole procedure.

#### The classic two-step trans-epithelial PTK-PRK

The first step of the procedure is epithelial removal after selecting the PTK mode in the EX-500 treatment planning section. Data entry included the pupillary diameter, thinnest pachymetry, the epithelial ablation depth and the optical zone (OZ). In all included eyes, the epithelium was removed using excimer laser (193 nm wave length) in a fixed ablation depth of 50 μm, an optical zone diameter of 7 mm and an ablation zone of 8.9 mm. The second step of the procedure is refractive correction, which necessitated switching to the wavefront-optimized mode in the EX-500 treatment planning section. Data entry included patient’s refraction and keratometry followed by excimer laser stromal ablation with a standard optical zone of 6.5 mm with an ablation zone of 7.1 mm in myopia and 9 mm in myopic astigmatism.

#### The new single-step trans-epithelial PRK

The StreamLight PRK software (WaveLight®; Alcon Laboratories, Ft Worth, TX, USA) incorporates the epithelial removal and excimer laser stromal ablation in one-step. Data entry included the patient’s refractive error, keratometry, pupillary diameter, thinnest pachymetry and the epithelial ablation depth (range from 45 to 65 μm) consistent with AS-OCT epithelial mapping. If the operator selected the standard 6.5 mm OZ for stromal ablation, the optical zone diameter for epithelial ablation will be automatically customized to 7 mm in myopia and 8 mm in myopic astigmatism with an ablation zone for both the epithelial and stromal ablation circles of 7.1 mm in myopia and 9 mm in myopic astigmatism. The stream treatment started with epithelial ablation and was interrupted (as recommended by the manufacturer) for few seconds to cool the cornea on hearing a 3-pop sound indicating the transition from the epithelial ablation to the stromal excimer laser ablation part. [Video 1] After completion of each procedure in both groups, Mitomycin C 0.02% was applied for 30 seconds followed by copious irrigation with cold balanced salt solution (BSS, Alcon Lab., Fort Worth, TX, USA). A soft bandage contact lens was applied until complete epithelial healing. Our Post-PRK treatment regimen included Moxifloxacin 0.5% eye drops four times daily for two weeks, Fluorometholone 0.1% eye drops twice daily for a month, preservative free lubricant eye drops five times daily for three months and oral non-steroidal anti-inflammatory pills twice daily for the first three postoperative days and once daily for the next three days to control post-PRK pain.

All surgical procedures in both groups were done by the same ophthalmic surgeon (M.A).

### Primary outcome measures

1. Visual Acuity: UDVA & CDVA were assessed using Snellen’s acuity chart converted to a logarithm of the minimum angle resolution (logMAR) at 1 week,1 month, 3 and 6 months after surgery.
2. Manifest refraction: Manifest sphere, cylinder and refraction spherical equivalent (MRSE) were measured with the same preoperative tool at 1 week, 1 month, 3 and 6 months after surgery.

### Secondary outcome measures

1. Epithelial healing: Patients were scheduled for daily follow up visits at the same time of the day until complete epithelial healing was confirmed by negative staining of the cornea using sterile fluorescein 2% strip (Medicare Inc., Mumbai, India).
2. Pain scoring: The Verbal Rating Scale (VRS) is a simple single-dimensional pain scoring scale utilized for post-PRK pain assessment in both groups [14]. The pain scores were recorded at 8 h, 1 day, 3 and 7 days following surgery by directly contacting the patients during their daily follow up visits until confirmed epithelial healing and by telephone thereafter. Patients were requested to evaluate their pain level and the doctor gave it a score from 0 to 4 (Zero for no pain, 1s for mild pain, 2 for moderate pain, 3 for severe pain and 4 for unbearable pain).
3. Haze scoring: Corneal haze was assessed at 1 month and 3 months following surgery using slit lamp and a score was given based on Fantes et al. scale [15].
  0 No haze, completely clear cornea
  0.5 Trace haze seen with careful oblique illumination
  1 Haze not interfering with the visibility of fine details of the iris
  2 Mild obstruction of iris details
  3 Moderate obstruction of the iris and lens
  4 Complete opacification of the stroma in the area of the scar, anterior chamber is totally obscured.

All post-operative outcome measures were assessed by the same ophthalmologist (M.O) being masked about the performed procedure (masked outcome assessor).

### Statistical analysis

Data were analysed using the Statistical Package for Social Science (SPSS, version 26.0, IBM Corp.). Qualitative data were expressed as frequency and percentage while quantitative data were tested for normality by Shapiro–Wilk test and expressed as Mean ± SD/SE (Standard deviation/Standard of error). Independent Sample *T*-test with equal variance was used to compare mean refractive outcomes between the two groups at each time point separately. One way repeated measures ANOVA test was used to identify changes over time within each group. Paired *T*-test was used to compare the first and the 6th month results in each group. Mann–Whitney *U* test was used to compare epithelial healing time and pain scores between the two groups and Chi-Square test for categorical haze grading. Spearman’s correlation was used to explore the correlation between the depth of stromal ablation in both groups and postoperative haze score at three months and to explore the correlation between the depth of epithelial ablation in the single-step TE-PRK group and the postoperative MRSE at six months.

The level of significance was considered at *P* value < 0.05.

The sample size was calculated using G power software version 3.1.3, using t test for comparison difference between two independent means, hypothesized effect size 0.5, alpha error probability 0.05, power (1- beta error probability) 0.80 and allocation ratio 1:1.

## Results

### Demographic data and preoperative baseline characteristics

The study included 108 eyes of 54 patients. Two patients in each group were lost to follow up and were excluded. The final sample was 100 eyes (25 patients/50 eyes in each study group). No significant differences were observed between the two groups regarding gender, mean age, UDVA, CDVA, manifest sphere, cylinder, MRSE, calculated ablation depth, the central epithelial thickness, Schirmer I test and TBUT as summarized in Table 1.

**Table 1:** Demographic data and preoperative baseline characteristics.

Parameter	2 Step PTK-PRK	Single Step TE-PRK	*p* value
No. of Eyes	50	50	–
No. of Patients	25	25	–
Age (years)^a^	25.1 ± 1.4	25.4 ± 1.9	0.912
Range	20–35	20–37	
Gender (Female/Male)	(16/9)	(17/8)	–
UDVA (logMAR)^a^	1.04 ± 0.19	1.03 ± 0.23	0.948
CDVA (logMAR)^a^	0.02 ± 0.006	0.02 ± 0.006	0.697
Manifest Sphere (D)^a^	−2.42 ± 0.18	−2.37 ± 0.25	0.548
Range	From 0 to −5	From 0 to −6	
Manifest Cylinder (D)^a^	−1.50 ± 0.16	−1.26 ± 0.14	0.329
Range	From 0 to −4	From 0 to −4	
MRSE (D)^a^	−3.17 ± 1.14	−2.99 ± 1.61	0.269
Range	From −1 to −5.5	From −0.75 to −6	
Central Epithelial Thickness (μm)^a^	52.68 ± 2.21	53.24 ± 1.82	0.096
Range	49–56	50–57	
Calculated depth of ablation (μm)^a^	61.76 ± 15.38	61.34 ± 23.37	0.694
Range	31–88	23–108	
Schirmer I test (mm)^a^	16.66 ± 0.9	16.42 ± 1.0	0.234
Range	15–18	15–19	
TBUT (seconds)^a^	12.04 ± 0.79	11.88 ± 0.79	0.275
Range	10 to 13	10 to 14	

Independent Sample *T-*test to compare means between the two groups.

*CDVA* Corrected distance visual acuity, *D* Dioptres, *logMAR* logarithm of the minimum angle resolution, *MRSE* Manifest refractive spherical equivalent, *PTK-PRK* Phototherapeutic keratectomy-photorefractive keratectomy, *TBUT* Tear film break-up time,*TE-PRK* Transepithelial photorefractive keratectomy, *UDVA* Uncorrected distance visual acuity.

^a^Data expressed as Mean ± SD/SE, Range.

### Refractive outcomes

Visual recovery was achieved significantly faster in the single-step PRK group. The mean postoperative UDVA was significantly better in the single-step PRK group than in the two-step PTK-PRK group (*p* < 0.001, independent sample *t*-test) at one week, one month, three and sixth months. At the end of the follow up period, thirty six eyes (72%) achieved UDVA of 20/20 and 46 eyes (92%) achieved UDVA of 20/25 or better in the single-step PRK group while in the two-step PTK-PRK group, eighteen eyes (36%) achieved UDVA of 20/20 and 39 eyes (78%) achieved UDVA of 20/25 or better. Meanwhile, the mean CDVA was statistically significant better in single-step PRK group at one week but there was no difference at one month, three and six months. The mean manifest sphere and MRSE showed a statistically significant difference at all follow up visits in favour of single-step PRK (*p* value < 0.001, independent sample *t*-test). Astigmatic efficacy analysis of both techniques showed significantly better outcome in the single-step PRK group compared with the two-step PTK-PRK group (*p* value < 0.001) at different follow up visits. In the single-step PRK group, the mean preoperative cylinder significantly improved from −1.26 ± 0.14 to −0.17 ± 0.03 and 39 eyes (78%) had a postoperative cylinder of 0.25 D or less at 6 months. In the two-step PTK-PRK group, the mean preoperative cylinder significantly improved from −1.50 ± 0.16 to −0.43 ± 0.02 and 16 eyes (32%) had a postoperative cylinder of 0.25 D or less at six months.

**Fig. 1:**
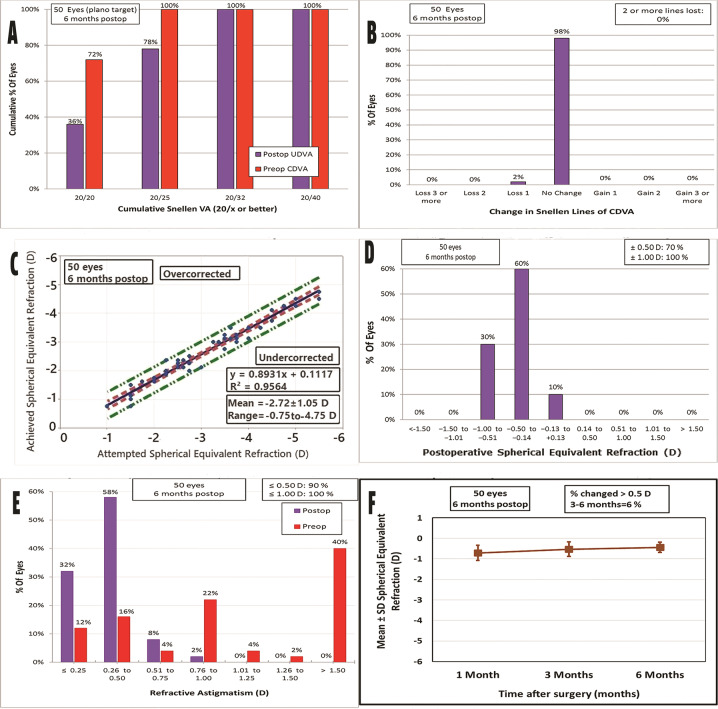
Standard graphs for reporting refractive outcomes for the two-step PTK-PRK group at sic months postop. **A** Uncorrected Distance Visual Acuity. **B** Change in Corrected Distance Visual Acuity. **C** Attempted vs Achieved Spherical Equivalent. **D** Spherical Equivalent Refractive Accuracy. **E** Refractive Astigmatism. **F** Stability of Spherical Equivalent Refraction. (CDVA corrected distance visual acuity, PTK-PRK Phototherapeutic keratectomy-photorefractive keratectomy, UDVA uncorrected distance visual acuity).

**Fig. 2:**
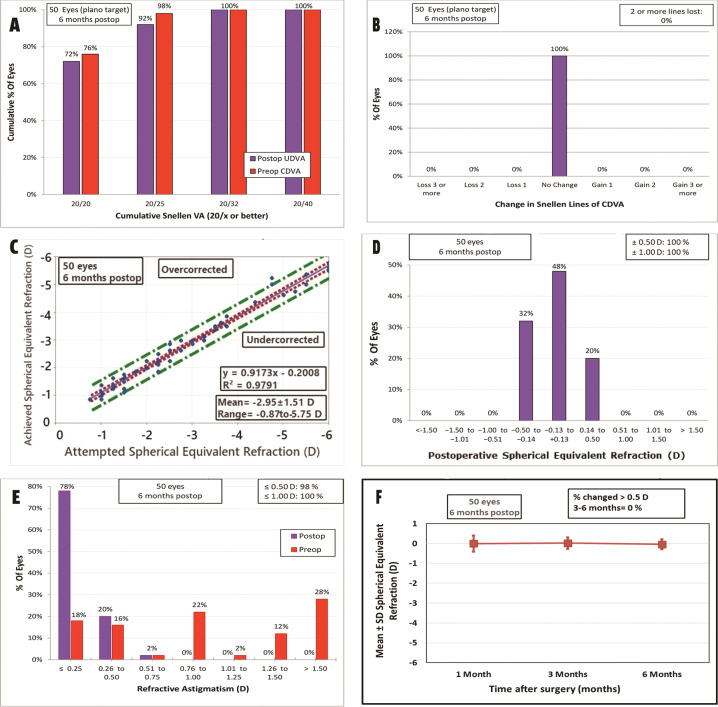
Standard graphs for reporting refractive outcomes for the single-step TE-PRK group at six months postop. **A** Uncorrected Distance Visual Acuity. **B** Change in Corrected Distance Visual Acuity. **C** Attempted vs Achieved Spherical Equivalent. **D** Spherical Equivalent Refractive Accuracy. **E** Refractive Astigmatism. **F** Stability of Spherical Equivalent Refraction. (CDVA corrected distance visual acuity, TE-PRK Transepithelial photorefractive keratectomy, UDVA uncorrected distance visual acuity).

Figures 1 and 2 show the six-standard graphs for reporting refractive outcomes for the two-step PTK-PRK and single-step TE-PRK groups respectively.

One-way repeated measure ANOVA test used to assess results in each group separately showed significant improvement of the mean UDVA (*p* value < 0.001) and the mean CDVA. All refractive parameters including the mean manifest sphere, cylinder and MRSE revealed a significant refractive correction in each group (*p* value < 0.001). Paired *T*-test used to compare the 1st month and 6th months results in each group showed a significant improvement (*p* value < 0.001) in all parameters in the PTK-PRK group except the CDVA, while no significant change was observed in single-step TE-PRK group.

Table 2 summarizes the postoperative refractive outcomes of the two transepithelial PRK techniques.

**Table 2:** Refractive outcomes, epithelial healing and haze grading of the two transepithelial PRK techniques.

Parameters	Two-step PTK-PRK (*n* = 50)	Single step TE-PRK (n = 50)	*P* value^a^
UDVA logMAR^b^			
Pre-operative	1.04 ± 0.19	1.03 ± 0.23	0.948
Postop. 1 week	0.30 ± 0.01	0.10 ± 0.01	<0.001^c^
Postop. 1 month	0.14 ± 0.01	0.04 ± 0.01	<0.001^c^
Postop. 3 months	0.10 ± 0.01	0.03 ± 0.009	<0.001^c^
Postop. 6 months	0.08 ± 0.01	0.03 ± 0.008	<0.001^c^
*P* value^d^	<0.001^c^	<0.001^c^	
*P* value^e^	<0.001^c^	0.25	
CDVA logMAR^b^			
Pre-operative	0.02 ± 0.006	0.02 ± 0.006	0.697
Postop. 1 week	0.06 ± 0.009	0.03 ± 0.008	0.036^c^
Postop. 1 month	0.03 ± 0.006	0.02 ± 0.007	0.459
Postop. 3 months	0.02 ± 0.006	0.02 ± 0.006	0.771
Postop. 6 months	0.03 ± 0.006	0.02 ± 0.006	0.645
*P* value^d^	<0.001^c^	0.01^c^	
*P* value^e^	0.3	0.3	
Manifest sphere (D)^b^			
Pre-operative	−2.42 ± 0.18	−2.37 ± 0.25	0.548
Postop. 1 week	−0.63 ± 0.05	0.08 ± 0.06	<0.001^c^
Postop. 1 month	−0.39 ± 0.04	0.10 ± 0.04	<0.001^c^
Postop. 3 months	−0.25 ± 0.04	0.10 ± 0.03	<0.001^c^
Postop. 6 months	−0.23 ± 0.03	0.04 ± 0.02	<0.001^c^
*P* value^d^	<0.001^c^	<0.001^c^	
*P* value^e^	<0.001^c^	0.1	
Manifest cylinder (D)^b^			
Pre-Operative	−1.50 ± 0.16	−1.26 ± 0.14	0.337
Postop. 1 week	−0.82 ± 0.04	−0.28 ± 0.05	<0.001^c^
Postop. 1 month	−0.64 ± 0.04	−0.21 ± 0.05	<0.001^c^
Postop. 3 months	−0.59 ± 0.04	−0.17 ± 0.03	<0.001^c^
Postop. 6 months	−0.43 ± 0.02	−0.17 ± 0.03	<0.001^c^
*P* value^d^	<0.001^c^	<0.001^c^	
*P* value^e^	<0.001^c^	0.337	
MRSE (D)^b^			
Pre-operative	−3.17 ± 1.14	−2.99 ± 1.61	0.269
Postop. 1 week	−1.03 ± 0.06	−0.05 ± 0.07	<0.001^c^
Postop. 1 month	−0.71 ± 0.05	−0.003 ± 0.05	<0.001^c^
Postop. 3 months	−0.53 ± 0.04	0.01 ± 0.04	<0.001^c^
Postop. 6 months	−0.44 ± 0.03	−0.04 ± 0.03	<0.001^c^
*P* value^d^	<0.001^c^	<0.001^c^	
*P* value^e^	<0.001^c^	0.218	
Epithelial healing days^b^			*P* value^f^
Mean ± SD	5.48 ± 0.76	3.24 ± 0.43	<0.001^c^
Range	(4.00–7.00)	(3.00–4.00)	
Median	5	3	
Haze grade (3 mo)	Eyes *n* (%)	Eyes *n* (%)	*P* value^g^
0	30 (60%)	31 (62%)	0.89
0.5	17 (34%)	17 (34%)	
1	3 (6%)	2 (4%)	

*CDVA* Corrected distance visual acuity, *D* Dioptres, *MRSE* Manifest refractive spherical equivalent, *PTK-PRK* Phototherapeutic keratectomy-photorefractive keratectomy, *TE-PRK* Transepithelial photorefractive keratectomy, *UDVA* Uncorrected distance visual acuity 1

^a^Independent Sample *T*-test to compare means between the two groups.

^b^Data expressed as Mean ± SD/SE.

^c^Statistical significance.

^d^One-way repeated measure ANOVA test within each group effect: preop., 1 week, 1- month, 3 and 6 months postop.

^e^Paired *T*-test to compare mean results between 1 month and 6 months follow up visits in each group.

^f^Mann–Whitney *U* test.

^g^Chi-Square test.

### Epithelial Healing

Epithelial healing was significantly faster in the single-step PRK group. The mean complete epithelial healing duration was 3.24 ± 0.43 and 5.48 ± 0.76 days in the single-step PRK and the PTK-PRK groups respectively (*p* value < 0.001) (Table 2). At the 3rd day follow up visit, thirty eight eyes (76%) showed complete epithelial healing in the single-step PRK group compared with zero eyes in the PTK-PRK group. Complete epithelial healing was documented in all included eyes of both groups on postoperative Day 7.

### Pain scoring

**Fig. 3:**
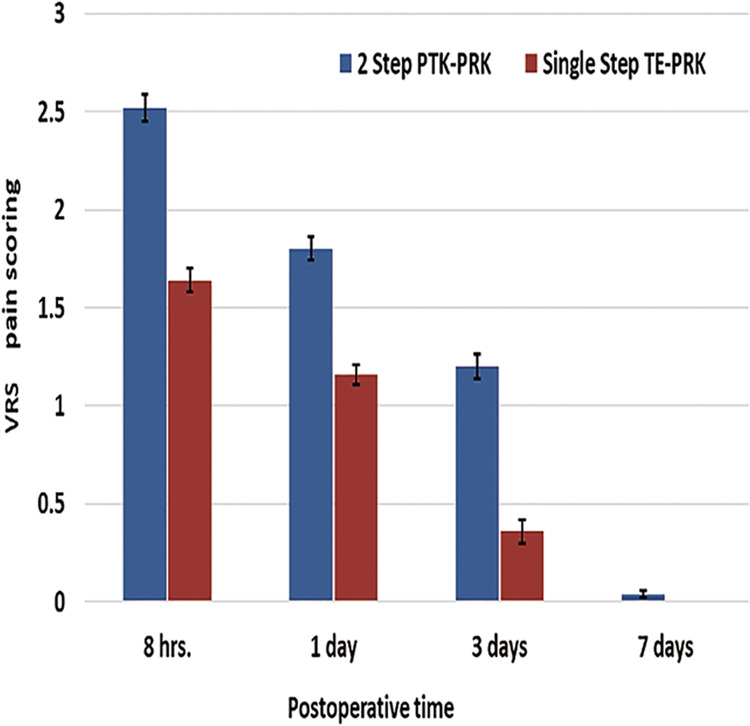
VRS pain scale of the two transepithelial PRK techniques. Comparison of the VRS pain scale between the two-step PTK-PRK and the single-step TE-PRK. (PTK-PRK Phototherapeutic keratectomy-photorefractive keratectomy, TE-PRK Transepithelial photorefractive keratectomy, VRS Verbal rating scale).

Evaluation of the subjective postoperative pain level according to the VRS revealed that the pain scores were significantly lower in the single-step TE-PRK group at 8 h, 1 day and 3 days after the PRK procedure (p value < 0.001) while at the 7th day follow up visit, there was no significant difference. The pain scores in both groups at different follow up visits are illustrated in Fig. 3.

### Postoperative Haze assessment

None of the included patients in both groups showed subepithelial haze exceeding grade 1 at both the first and 3rd month follow up visits. Haze scores showed no statistical difference between the two groups at the 3rd month follow up visit. Thirty one eyes (62%) in the single-step TE-PRK group had zero haze compared with 30 eyes (60%) in the PTK-PRK group as illustrated in Table 2.

### Postoperative complications

No postoperative complications were encountered in both studied groups apart from the previously analysed pain and haze.

### Correlations

#### Correlation between the depth of stromal ablation and postoperative haze

A significant positive correlation was observed between the calculated depth of stromal ablation in all eyes enrolled in the study in both transepithelial PRK groups and the postoperative haze at three months follow up (*r* = 0.48, *p* value < 0.001).

#### Correlation between the depth of epithelial ablation in single-step TE-PRK group and the postoperative MRSE

A positive correlation between the depth of epithelial ablation in single-step TE-PRK group and the postoperative MRSE at the end of follow up period (six months) was observed but it was of no statistical significance (*r* = 0.27, *p* value = 0.054)

## Discussion

Epithelial removal in PRK could be performed manually, alcohol assisted or with the use of excimer laser [16]. Epithelial removal using excimer laser could provide a more uniform epithelial ablation compared with conventional methods thus resulting in faster epithelial healing with less pain and haze as suggested by Fadlallah et al. [12].

Naderi et al. [17] and Gaeckle [18] compared single-step TE-PRK and mechanical PRK and concluded that single-step TE-PRK offered faster visual recovery, epithelial healing and less pain compared with manual PRK. Aslanides et al. [19] also reported that single-step TE-PRK provided faster epithelial healing, lower pain scores, and significantly less haze formation compared with alcohol assisted PRK.

To the best of our knowledge, this is the first study to compare the two available trans-epithelial PRK techniques, the classic two-step transepithelial PTK-PRK and the new single-step transepithelial PRK.

Computer generated randomization was utilized to avoid selection bias. Preoperative data were similar in both groups with no significant difference regarding the demographic, visual and refractive parameters. The surgical nomogram and the postoperative treatment regimen were identical in both groups. Masking of the outcome assessor was also applied.

Accurate measurement of the epithelial thickness using AS-OCT provides a more precise way to ensure complete epithelial removal thus avoiding inexact stromal ablation with resultant under or overcorrection. Epithelial thickness in our study was measured using Spectralis OCT epithelial mapping in both groups.

One of the major disadvantages of the transepithelial two-step PTK-PRK technique in EX-500 is that if the epithelial ablation depth in PTK mode exceeds 50 μm, the software limits the epithelial optical zone diameter to 5.0 mm resulting in a non-perfect match between the epithelial and the stromal ablation zones. This disadvantage has been overcome in the single-step TE-PRK (StreamLight software) where the epithelial ablation depth can be adjusted in a range from 45 to 65 μm without affecting the epithelial optical zone diameter.

Dupps et al. [20] documented hyperopic shifts in postoperative MRSE with deeper PTK-type epithelial ablations exceeding 50 μm with larger optical zone compared with lower PTK-type ablations of 50 μm with smaller optical zone. Therefore, we explored a correlation between the depth of epithelial ablation and the postoperative MRSE in the single-step TE-PRK group at the end of follow up period of the present study but there was no significant correlation.

Although both TE-PRK groups showed significant improvement of postoperative refractive outcome parameters (postoperative UDVA, the manifest sphere, cylinder and MRSE at all follow up visits), a faster visual recovery with a better refractive outcome was achieved in the single-step TE-PRK group when compared with the PTK-PRK group. On comparing the first and the 6th month refractive results in each group, there was no significant change in the single-step TE-PRK group reflecting stability of refractive outcome while there was a significant improvement in the two-step PTK-PRK group.

The superior refractive outcome in the single-step TE-PRK group in the current study has various interpretations. First, the more accurate matching between the epithelial and stromal ablation zones with one centration applicable throughout the whole procedure. Secondly, the unique epithelial ablation profile in StreamLight PRK that utilizes more pulses outside the 4 mm zone to prevent peripheral epithelial remnants but not deep enough to reach the stroma in the periphery that would induce a myopic shift. Thirdly, the shortened surgical time avoiding excessive dehydration. Finally, the software permitting the entry of an accurate epithelial depth exceeding 50 μm if needed compared with a fixed depth of 50 μm in the two-step PTK-PRK that may leave epithelial remnants explaining the observed postoperative under-corrections.

No intra or postoperative complications apart from pain and haze were reported in our study. Post-PRK pain scores were less in the single-step TE-PRK group initially but later scores were similar at the 7th day. Kaluzny et al. [11] in a different comparative study found no differences in pain intensity between single-step TE-PRK and alcohol assisted PRK but they admitted that there was a statistical gender difference with more females in TE-PRK group.

None of our patients experienced haze exceeding grade 1 at the first and 3rd month follow up visits in both groups. Gadde et al. [13] compared corneal haze in a different comparative study between manual PRK and single-step TE-PRK and reported more trace haze in TE-PRK group but the degree of myopia was higher in the single-step TE-PRK group. In addition, they used a different device (Amaris excimer laser, version 500 E Schwind/eye‑tech‑solutions).

A positive significant correlation between the stromal ablation depth in all eyes of both groups and postoperative PRK haze was observed that is consistent with Møller-Pedersen et al. [21] and Spadea et al. [22] who found that the development and duration of corneal haze after PRK increased proportionally with increasing stromal ablation depth.

Our study had some limitations such as the small sample size and lack of enough prior research to compare our results with. However, the high statistical difference between the two studied groups could enhance the external validity of the study. Other limitations include the inability to apply a contralateral eye study design, enrolling both eyes of each patient instead of randomly selecting one eye and the fact that the study was performed using one machine (WaveLight EX500, Alcon lab.). One of the limitations of the StreamLight PRK software is that it has many default settings especially related to optical zone parameters that can’t be adjusted freely.

In conclusion, both transepithelial PRK techniques are safe and effective for correcting mild to moderate degrees of myopia and myopic astigmatism, the superiority of single-step PRK could be attributed to the faster visual recovery achieved, the shorter epithelial healing time observed and the less pain experienced by patients. A study with a larger sample size comparing various transepithelial PRK techniques in different machines would provide more information.

## Summary

### What was known before

- Conventional PRK whether with mechanical or alcohol assisted epithelial removal is a well-established refractive procedure for correction of mild to moderate myopia or myopic astigmatism.
- Many studies compared the trans-epithelial and the conventional PRK procedures and suggested superiority of TE-PRK but no studies compared the available transepithelial PRK techniques.

### What this study adds

- The first study to compare the two transepithelial-PRK techniques; the 2 step PTK-PRK versus the new single step PRK.
- Single step TE-PRK achieved a faster visual recovery and a better refractive outcome with less post-PRK pain.

## Supplementary information

Video 1

Video Legends

## Data Availability

All data are available from the corresponding author upon a reasonable request.
