# Supplementary material for: Transepithelial photorefractive keratectomy: a prospective randomized comparative study between the two-step and the single-step techniques
Source: Eye (Lond). 2022 Jul 21;37(8):1545–52. doi: 10.1038/s41433-022-02174-4 (PMC10219954; doi:10.1038/s41433-022-02174-4)
Supplement: Supplementary file 2 — Video Legends [file 41433_2022_2174_MOESM2_ESM.docx]

**Video Legends:**

**Video [1]:** Single-step transepithelial photorefractive keratectomy (TE-PRK) surgical steps.
